# Clinical epidemiological analysis of cohort studies investigating the pathogenesis of kidney disease

**DOI:** 10.1007/s10157-021-02121-9

**Published:** 2021-08-10

**Authors:** Shigeru Tanaka, Toshiaki Nakano, Kazuhiko Tsuruya, Takanari Kitazono

**Affiliations:** 1 Department of Medicine and Clinical Science, Graduate School of Medical Sciences, Kyushu University, 3-1-1 Maidashi, Higashi-ku, Fukuoka, 812-8582 Japan; 2 Department of Nephrology, Nara Medical University, Nara, Japan

**Keywords:** Epidemiology, Causal effect, Observational study

## Abstract

In recent years, large cohort studies of patients with chronic kidney disease (CKD) have been established all over the world. These studies have attempted to analyze the pathogenesis of CKD using a large body of published evidence. The design of cohort studies is characterized by the measurement of the exposure prior to the occurrence of the outcome, which has the advantage of clarifying the temporal relationship between predictors and outcomes and estimating the strength of the causal relationship between predictors and multiple outcomes. Recent advances in biostatistical analysis methods, such as propensity scores and risk prediction models, are facilitating causal inference using higher quality evidence with greater precision in observational studies. In this review, we will discuss clinical epidemiological research of kidney disease based on the analysis of observational cohort data sets, with a focus on our previous studies.

## Introduction

In general, epidemiology is defined as a scientific method for clarifying the relationship between disease events and the factors that influence them, and for establishing effective countermeasures against health-related problems. There are two types of epidemiological studies: observational studies, in which the relationship between factors and diseases is clarified by observation alone without artificial manipulation, and intervention studies, in which changes in the occurrence and prognosis of diseases before and after artificial manipulation are experimentally confirmed. It should be noted that the results obtained in observational studies cannot completely rule out the possibility of an apparent relationship arising owing to chance, bias, or confounding [1–3]. In cohort studies, the temporal relationship between the exposures and the onset of the disease event is clear, making it possible to infer causality. Therefore, the evidence level of cohort studies ranks higher than that of other study designs, such as case–control and cross-sectional studies.

Cohort studies can identify risk factors for disease by observing a population for a period and comparing differences in the incidence of disease events with and without exposure. Bias and confounding can be removed to a certain extent through statistical analysis, but a randomized controlled intervention trial (RCT) is required to completely remove the effects of bias and confounding. However, there are many barriers to the implementation of RCTs, such as cost and ethical concerns. Evidence from well-designed cohort studies should, therefore, form the basis for inferring interventional factors that are predicted to have a causal effect on disease. The pathogenesis of chronic kidney disease (CKD) involves a wide range of factors, including immunological and environmental factors and genetic predisposition, and these interact with each other. To clarify the prognostic factors of CKD, it is important to create a systematic database comprising the vast amount of clinical information generated in daily practice. By creating such a database, it will be possible to clarify factors that are unclear in individual cases alone and to elucidate the mechanisms of CKD onset and exacerbation. To extract high-quality evidence from observational databases that can be used in disease prevention and intervention studies, it is necessary to apply appropriate methods of statistical analysis that control confounding and lead to accurate estimates. In addition to the current status of CKD cohorts around the world, herein, we review biostatistical methods that are useful in clinical research to establish evidence that can be used to elucidate the pathogenesis of CKD and improve medical practice.

## Representative non-dialysis-dependent CKD cohort studies

### Cohort studies of non-dialysis-dependent CKD outside Japan

#### African American Study of Kidney Disease and Hypertension (AASK)

The AASK cohort study was a follow-up study of an RCT conducted among 1094 black patients with CKD, with three initial antihypertensive drugs (ramipril, metoprolol, and amlodipine) and two antihypertensive targets (mean blood pressure 102–107 mmHg and < 92 mmHg) [4, 5]. Participants comprised 691 consented patients out of 787 who did not develop end-stage kidney disease (ESKD) during the AASK study period and were followed for up to 6.4 years. The black population in the United States is at high risk for worsening kidney function, because they are more susceptible to hypertension and kidney impairment than Whites and have a higher incidence of lifestyle-related diseases, such as obesity. This follow-up study showed an association between apolipoprotein L1 (APOL1) gene polymorphism and worsening kidney function [6, 7]. Recently, metabolomic analysis using frozen serum samples has also shown the association of specific serum metabolites with urinary protein and total mortality [8, 9].

#### Kidney Early Evaluation Program (KEEP)

The KEEP study is focused on improving the prognosis of kidney disease by increasing public awareness about the concept of kidney disease and encouraging early medical intervention in high-risk patients. This study was originally initiated by the National Kidney Foundation in 2000 to raise awareness about CKD screening. The target population is the general population aged 18 years and older who have hypertension, diabetes, or a family history of hypertension, diabetes, or kidney disease. Screening tests that include items on social background, pre-existing medical conditions, blood pressure measurement, and blood and urine tests are conducted, and participants with abnormal values are encouraged to visit a medical institution. Follow-up data after medical care are not included but can be merged with external data such as the National Death Index, the United States Renal Data System, and the National Health and Nutrition Examination Survey (NHANES). By cross-checking with external data sources, the association between demographic and other data such as body mass index, racial differences, health insurance status, total mortality, dialysis initiation, and cardiovascular disease can be examined [10–14].

#### Chronic Renal Insufficiency Cohort (CRIC)

The CRIC study is a prospective cohort study initiated by the National Institute of Diabetes and Digestive and Kidney Diseases in 2001 to investigate risk factors for the progression of kidney disease and the development of CVD, and to gain useful knowledge for future intervention studies. This study has been ongoing for more than 20 years from Phase I to Phase IV in a total of 3612 patients with CKD attending seven renal care centers. In addition to the basic study, many ancillary studies have been conducted and many articles have been published in medical journals. Since its inception, the CRIC has been strategically collecting data related to the care of patients with CKD, and biospecimen storage has been thorough. Over the past decade, the CRIC study has contributed substantially to our understanding of factors associated with CKD progression. Hannan et al. summarized findings from a longitudinal study that assessed risk factors associated with CKD progression in the CRIC study under six themes, as follows: (1) socioeconomic factors (sex, race/ethnicity, nephrological care); (2) behavioral factors (healthy lifestyle, diet, sleep); (3) genetic factors (APOL1, genome-wide association studies [GWAS], renin–angiotensin–aldosterone pathway genes); (4) cardiovascular (atrial fibrillation, hypertension, vascular stiffness); (5) metabolic (fibroblast growth factor 23 and urinary oxalate); and (6) novel factors (biomarkers of acute kidney injury and kidney impairment) [15]. The current fourth phase, which began in 2018, is increasing the ethnic diversity of the cohort by recruiting 500 Native American and 126 Hispanic adults. In addition, data collection has been refocused on incorporating new mobile technologies to remotely collect kidney and cardiovascular data from participants’ homes, further highlighting the importance of interdisciplinary collaborative opportunities.

#### Chronic Kidney Disease in Children (CKiD)

The cohort was established in 2006 and has enrolled and prospectively followed more than 800 pediatric patients with CKD from 57 sites across the United States. Patients between the ages of 1 and 16 years with an estimated glomerular filtration rate (GFR) of 30–75 mL/min/1.73 m^2^, not including those receiving dialysis, were included in the study. In addition to examining risk factors for progression of kidney failure, the study is unique in that it addresses research questions specific to pediatric practice, such as the impact of kidney function on growth and neurological development, the relationship between stunting and mortality risk, and epidemiological studies of pediatric cardiac disease [16].

#### German Chronic Kidney Disease (GCKD) study

The GCKD study is a prospective, observational, nationwide cohort study established in 2010. The study aims to enroll 5000 patients with CKD of various etiologies undergoing nephrological treatment and to follow them for up to 10 years. Patient recruitment and follow-up are being organized by collaborating centers with nephrologists throughout the country through an academic nephrology network. Patients with an estimated GFR between 30 and 60 mL/min/1.73 m^2^ at the time of enrollment, or with overt proteinuria if GFR is > 60 mL/min/1.73 m^2^, are included. Biomaterials such as DNA, serum, plasma, and urine are collected using standardized methods to identify biomarkers associated with CVD incidence and CKD progression [17]. In addition to epidemiological topics such as blood pressure [18], heart failure [19], dietary patterns [20], and gout [21], biomarkers and genes such as urine 6-bromotryptophan [22], serum uromodulin [23], GWAS of urate and gout [24], telomere length [25], and other biomarkers and genes have been actively investigated.

### Cohort studies of non-dialysis-dependent CKD in Japan

#### Chronic Kidney Disease Japan Cohort (CKD-JAC)

The CKD-JAC study was established in 2007 to determine the incidence of CVD, ESKD, and all-cause mortality in the Japanese population. The CKD-JAC is a 4-year prospective observational cohort of patients attending nephrology centers. Inclusion criteria are (1) Japanese and Asian patients living in Japan, (2) age 20–75 years, and (3) estimated GFR 10–59 mL/min/1.73 m^2^ [26]. To date, 15 studies have been published on CVD incidence, sleep, hospitalization events, and progression factors of left ventricular hypertrophy.

#### Gonryo study

The Gonryo study is a 5-year prospective cohort of 2692 outpatients with CKD from 11 centers that began in 2007. All patients met the criteria for CKD and had a persistently low GFR of less than 60 mL/min/1.73 m^2^ or proteinuria by urinalysis [27]. To date, six studies have reported on CVD incidence, anemia, blood pressure, and CKD progression.

#### Fukuoka Kidney disease Registry (FKR) study

The FKR is a prospective, multicenter, observational cohort of patients with CKD before dialysis that began enrollment in 2013. Approximately 4500 patients were enrolled in this study, with extensive lifestyle surveys and collection of biological samples including blood, urine, and DNA at baseline enrollment, followed by a planned 5-year follow-up, which is still underway [28].

The objectives of this cohort are as follows:

I. To identify and validate biomarkers, including novel risk factors and genetic susceptibility, using biological materials and high-throughput omics technology;
II. To clarify the effects of genetic and environmental interactions on the risk of adverse events in Japanese patients with CKD;
III. To evaluate the impact of lifestyle issues such as sex, nutrition, exercise, quality of life (QOL), and psychological and socioeconomic factors on CKD progression and complications; and
IV. To assess the socioeconomic burden of age-related complications and health resource utilization in older patients with CKD.

Together with the search for risk factors in epidemiological studies and multi-layered omics analysis, the findings of our cohort study will elucidate the molecular mechanisms that determine the phenotype of the disease and will contribute to personalized medicine and healthy longevity in patients with CKD.

## Representative hemodialysis cohort studies

### Hemodialysis cohort studies outside Japan

#### Dialysis Outcomes and Practice Patterns Study (DOPPS)

The DOPPS is a large, international observational study of practice patterns and patient outcomes in patients receiving maintenance hemodialysis. The data collected in each country will be sent for analysis to the Arbor Research Collaborative for Health, a non-profit research organization in the United States. The study sites will be randomly selected to be representative of dialysis facilities in each participating country, taking into account facility type and geographic distribution. The DOPPS is collecting data on four themes: (1) life expectancy, (2) hospitalization, (3) vascular access, and (4) QOL. It has been shown that mortality is largely explained by regional differences in survival (especially when comparing the United States and Europe) and by differences in the use of vascular access in facilities [29, 30]. The DOPPS also showed that longer treatment time was associated with lower mortality in models adjusted for Kt/V and other characteristics using both standard and instrumental variable analysis methods [31].

### Hemodialysis cohort studies in Japan

#### Japan Dialysis Outcomes and Practice Patterns Study (J-DOPPS)

The J-DOPPS, a sub-cohort of the DOPPS, is a prospective observational study of Japanese patients receiving maintenance hemodialysis. J-DOPPS analysis has reported a better life expectancy for patients with maintenance hemodialysis in Japan than in the United States and Europe, and a higher use of arteriovenous fistulas in Japan [29, 30]. Recently, it has been shown that the rate of adherence to treatment plans (frequency and duration) and the number of physician visits are much higher in Japan than in Europe and the United States [32]. The wide range of new findings from the J-DOPPS will have a great impact on dialysis treatment in Japan and is expected to contribute to further improvement of patient outcomes.

#### Japanese Society for Dialysis Therapy Renal Data Registry (JRDR)

The JRDR is a database based on an annual survey conducted by the Japanese Society for Dialysis Therapy (JSDT), which has accumulated data on approximately 870,000 patients undergoing dialysis. In the past, reference values for guidelines have been formulated on the basis of the results of analyses of this database. In recent years, a number of important studies have emerged from this database that will have an important impact on dialysis care in Japan, including a study that showed an association between pre-dialysis serum magnesium concentration and total mortality, cardiovascular death, and non-cardiovascular death at 1 year [33], and a study that reported an association between hyponatremia and total mortality and cardiovascular risk [34].

#### Kyushu Prospective Cohort Study in Hemodialysis Patients (Q-Cohort Study)

We have conducted the Q-Cohort Study since 2006, a prospective cohort study of approximately 3600 patients on chronic hemodialysis, with outcomes including all-cause mortality, death from infection or tumor, major adverse cardiovascular events (MACE), and new fractures. The clinical background of included patients is very similar to that of Japanese patients undergoing dialysis as of December 31, 2006, as compiled and published by the JSDT, except for their longer dialysis time. To date, the following studies have been conducted: hypo-responsiveness to erythropoiesis-stimulating agents and increased risk of total mortality and MACE [35], use of vitamin D receptor agonists (VDRA) and decreased risk of infectious disease mortality [36], hyperphosphatemia and increased risk of cerebral hemorrhage [37], increased cardiothoracic ratio and increased risk of total mortality and MACE [38], muscle mass loss and increased risk of fracture [39], treatment-resistant hypertension [40], and multi-vascular disease and cardiovascular prognosis [41]. We are currently conducting a follow-up study to collect additional information such as cognitive function and frailty in a new target population by establishing a second population starting in 2019. The characteristics of the cohort studies described in this manuscript are shown in Table 1.

**Table 1:** The representative cohort study of patients with chronic kidney disease

Study	Participants	Renal function, mL/min/1.73 m^2^	Number of enrolled patients	Biospecimen available	Geographic region	References [Citation numbers in the manuscript]
Cohort studies of non-dialysis dependent CKD patients						
African American Study of Kidney Disease and Hypertension (AASK)	African Americans, ages 18–70, diastolic blood pressure more than 95 mm Hg	GFR 20–65	1094	Buffy coat, serum, urine	United States	Lea et al. [4], Wright et al. [5]
Kidney Early Evaluation Program (KEEP)	Adults with 18 years old or older, history of diabetes mellitus or hypertension, or a family history of kidney disease, diabetes mellitus, or hypertension	GFR < 60	16,129	–	United States	McCullough et al. [10], Myers et al. [11], Jolly et al. [12], Babayev et al. [13], Jurkovitz et al. [14]
Chronic Renal Insufficiency Cohort (CRIC)	Racially diverse, ages 21–74	eGFR 20–70	3612	Blood, urine	United States	Hannan et al. [15]
Chronic Kidney Disease in Children (CKiD)	Children, ages 1–16 years	eGFR 30–90	830	Blood, urine	United States	Furth et al. [16]
German Chronic Kidney Disease (GCKD) study	Caucasian, ages 18–74	eGFR 30–60 or overt proteinuria and eGFR > 60		Blood, urine	Germany	Eckardt et al. [17]
Chronic Kidney Disease Japan Cohort (CKD-JAC)	Japanese and Asians living in Japan, ages 20–75	eGFR 10–59	3084	Plasma, urine	Japan	Imai et al. [26]
Gonryo study	Japanese outpatients with CKD	–	4015	–	Japan	Yamamoto et al. [27]
Fukuoka Kidney disease Registry (FKR) study	Japanese CKD patients, ages 18 years and older	eGFR < 60 or eGFR ≥ 60 with any kidney damage	4476	Plasma, serum, urine	Japan	Tanaka et al. [28]
Cohort studies of hemodialysis patients						
Dialysis Outcomes and Practice Patterns Study (DOPPS), Japan Dialysis Outcomes and Practice Patterns Study (J-DOPPS)	CKD patients receiving hemodialysis, ages 18 years and older	Hemodialysis	2169	–	Global	Goodkin et al. [29], Pisoni et al. [30], Tentori et al. [31], Saran et al. [32]
Japanese Society for Dialysis Therapy Renal Data Registry (JRDR)	Dialysis patients aged 18 years and older, undergoing any type of renal replacement therapy (nearly all dialysis patients in Japan)	Hemodialysis	273,097	–	Japan	Sakaguchi et al. [33], Fujisaki et al. [34]
Kyushu Prospective Cohort Study in Hemodialysis Patients (Q-Cohort Study)	Outpatients aged 18 years and older, who underwent regular hemodialysis therapy	Hemodialysis	3598	–	Japan	Eriguchi et al. [35], Tanaka et al. [36, 40, 41], Yamada et al. [37, 39], Yotsueda et al. [38]

*CKD* chronic kidney disease; *eGFR* estimated glomerular filtration rate

## Biostatistical methods applicable to cohort data

To extract high-quality evidence from observational databases, it is necessary to apply appropriate methods of statistical analysis that control for confounding and lead to precise estimates. Currently, the search for novel disease risk markers is progressing, and the trend in clinical research is to create risk models that incorporate risk markers to stratify specific populations and test the efficacy of personalized treatment based on accurate individual risk prediction. In the following sections, we will outline the biostatistical methods that we have used and the evidence we have derived using these methods.

### Risk prediction model

**Fig. 1:**
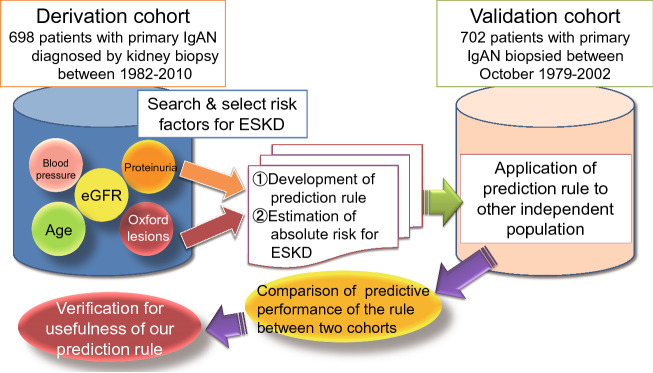
Brief summary of study design. Abbreviations: *IgAN* immunoglobulin A nephropathy; *eGFR* estimated glomerular filtration rate; ESKD, end-stage kidney disease

Clinical decisions regarding the diagnosis and treatment of individual patients are routinely determined under the constraint of various uncertainties. Therefore, obtaining accurate predictions of clinical prognosis is critically important for patient management of any disease. We developed and validated a clinical pathological prediction rule to calculate the absolute risk of long-term kidney prognosis in a large cohort of patients with immunoglobulin A (IgA) nephropathy [42]. The prediction rule can stratify patients according to the probability of future events by scoring clinical findings and the results of diagnostic tests. Since it is obvious that the internal validity of the prediction model generated by the derivation sample is high, the external validity of the model needs to be proved by testing the goodness of fit to another independent population (validation sample). Figure 1 shows brief summary of this study design. The prediction rule we created had nearly equivalent predictive performance for kidney prognosis in two independent cohorts (generation cohort, *n* = 698; validation cohort, *n* = 702), demonstrating its usefulness for stratifying the risk of developing ESKD using absolute risk in individual cases. Recently, Ueki et al. developed a risk model for the development of CVD consisting of risk factors such as age, history of CVD, diabetic nephropathy, history of dialysis, serum albumin concentration, and urinary protein at 1 year after transplantation in a cohort of kidney transplant recipients; the model showed good external validity in an independent external cohort [43]. Such prediction rules to guide initial treatment decisions will contribute to the realization of personalized medicine through validation in future prospective observational studies and clinical trials.

### Risk reclassification

In recent years, the development of high-throughput measurement technologies has led to comprehensive exploratory studies of candidate compounds of interest in drug discovery and biomarker discovery research. The process of statistical validation is essential for the identification of new risk markers that can be applied to clinical practice. It is not enough for a novel risk marker to be an independent contributor to risk; it must also provide additional value in risk prediction over known and established risk markers. In other words, the relevance of a new risk marker should be assessed as an indicator of improved predictive performance of the risk model. The following criteria have been proposed for the evaluation phase of novel risk markers [44]: (i) discovery of novel marker; (ii) validation in a prospective cohort; (iii) confirmation of improved risk prediction; (iv) validation of clinical utility; (v) confirmation of improved clinical prognosis in interventional studies; and (vi) improvement in medical economy.

**Fig. 2:**
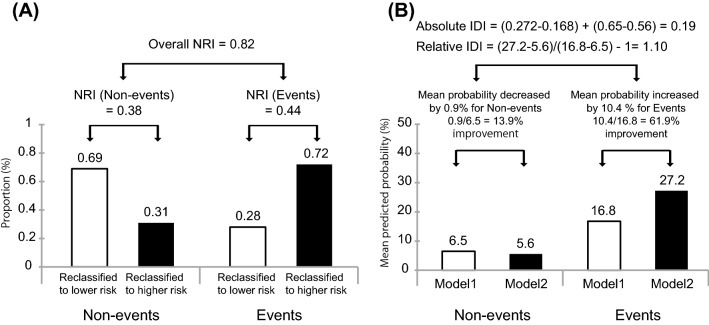
Graphical representation of NRI (**A**) and IDI (**B**) for disease events. The NRI plot shows the proportion of individuals reclassified to higher or lower risk after the addition of biomarkers to the clinical model. The IDI plot shows the mean predicted probability of disease events according to the prior (Model1) and novel (Mode2) models

Receiver Operating Characteristic (ROC) curve, net reclassification improvement (NRI), and integrated discrimination improvement (IDI) are frequently used as indices to evaluate the improvement of risk prediction ability of models in observational studies. The ROC curve is a two-dimensional graph that represents the discriminatory performance of a diagnostic test. The curve is drawn as a line plotting the true positive rate and false positive rate for each cutoff point that distinguishes abnormal from normal. The area under the ROC curve is called area under the curve (AUC), and an AUC value close to 1 means that the discriminatory ability of the diagnostic test is high. Reclassification is a statistical technique that examines how many patients can be reclassified by adding a new test or biomarker to an existing model for predicting the probability of the presence of a disease event. NRI can give clinical information about quantitative improvements by adding new biomarkers to traditional models. IDI can show that the addition of a new biomarker can improve sensitivity without sacrificing specificity. Therefore, the NRI and IDI are more sensitive than the AUC of ROC analysis, which shows improved predictive value [45, 46]. Figure 2 shows how Model 1 (existing model) is compared to Model 2 (new model) to calculate NRI and IDI values using the percentage of the population that has been reclassified.

We investigated whether serum bilirubin could be a biomarker for the development of ESKD in an IgA nephropathy cohort [47]. In this study, we found that serum bilirubin is an independent risk factor for the development of ESKD, and the C-statistic of the model incorporating bilirubin level was improved compared with the conventional model using only risk factors. Evaluation using net reclassification improvement and integrated discrimination improvement also showed a significant improvement in risk discrimination. Such risk model evaluation emphasizes the value of serum bilirubin as a potential biomarker for predicting kidney prognosis in patients with IgA nephropathy.

### Propensity score and instrumental variable methods

Covariate adjustment using the propensity score is useful to estimate the causal effect of treatment in observational studies where random assignment is not made and various types of confounding are likely to occur. The propensity score is the conditional probability of receiving the treatment rather than the control, given the observed covariates [48]. Specific adjustment methods using propensity scores include matching, stratification, analysis of covariance and inverse probability of treatment weighting (IPTW). The advantages and disadvantages of each statistical method are shown in Table 2. We used IPTW and instrumental variable methods to evaluate the effect of VDRA on infection-related mortality in a cohort of approximately 3500 hemodialysis patients (Q cohort study) [36]. We found a significant reduction in the risk of infection-related mortality in the intravenous group (*n* = 492) compared with the untreated group (*n* = 1007), and similarly, a significantly lower risk of mortality in the intravenous group compared with the oral group (*n* = 1878).

**Table 2:** Comparative characteristics of causal analysis methods

Analysis methods	Advantages	Disadvantages
Conventional covariate adjustment	Provides prognostic model for outcome of interest	Some confounders may not remain unbalanced between groups May not be suitable for studies with small sample sizes and many covariates
PS methods (Overall)	No need to set up regression models for covariates and dependent variables A large number of covariates can be reduced to a one-dimensional PS Robustness to model misconfigurations	Unable to control for unknown or unmeasured confounding factors
PS-matching	Easily understandable in presenting and interpreting analysis results All variables of interest are well balanced across comparison groups Estimates the average treatment effect for patients who received typical treatment	Selection criteria for matching pairs is arbitrary Data of subjects who are not selected as a pair is wasted Standard errors of causal effect estimates cannot be calculated accurately
Analysis of covariance using PS as a covariate	Retains data from all study participants	Assumption of a linear relationship between PS and the dependent variable
PS-stratification	Retains data from all study participants Can provide effect estimates for each stratum	When the number of stratums is large, the estimation accuracy is poor, especially for data sets with few outcomes Standard errors of causal effect estimates cannot be calculated accurately
IPTW	Retains data from all study participants Creates a pseudo population with perfect covariate balance	Unstable when extreme weights occur
Instrumental variable	Causal effects can be accounted for measured as well as unmeasured factors	Difficult to find a strong instrumental variable in addressing many assumptions
Randomized control trials	All variables of interest, including measured as well as unmeasured factors, can be perfectly balanced across comparison groups	High barriers in terms of cost, effort, and ethics

*PS* propensity score; *IPTW* inverse probability of treatment weighting

**Fig. 3:**
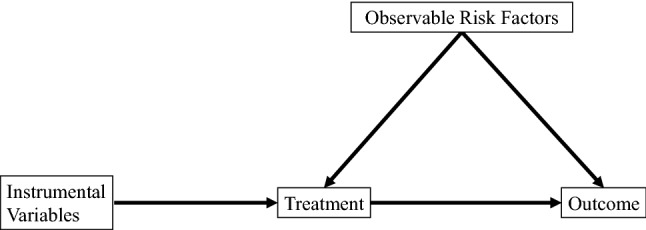
Schematic representation of the Instrumental variable method

A limitation of the propensity score method is that the effects of unmeasured or unknown confounders cannot be adjusted. The best way to adjust for unmeasured confounders is to conduct RCTs; however, the ethical and cost burdens of conducting such studies make it difficult to implement in practice. Therefore, in recent years, the adjustment of unmeasured factors using the instrumental variable method has been attempted. An instrumental variable is a factor that is associated with the exposure but not with the outcome [49]. The objective of the instrumental variable, which affects the treatment group independently of the observed values, is to mimic the randomization of subjects into the treatment group. A schematic representation of the Instrumental variable method is shown in Fig. 3. In the study described above, we applied both propensity score analysis and instrumental variable methods to test the robustness of the analysis results. The results showed that the results differed depending on the incorporation of nutritional indicators into the model, suggesting that residual confounding related to nutrition may have affected the treatment effectiveness of VDRA.

## System for promoting cohort studies

The most important points in promoting cohort studies are the establishment of a research organization and the development of human resources. The overall direction of the research analysis must be controlled under the supervision of the steering committee, which deliberates on the project plan, ethics, and validity of the study. The biostatistics team scientifically deliberates on the selection of analysis methods and the validity of results and supports the quality of the study in terms of research design and analysis. A clinical research coordinator who is in charge of the field research that forms the basis of the research, such as research registration and follow-up, is an important element in controlling the accuracy of cohort studies [28].

The involvement and training of researchers with expertise in clinical epidemiology research is essential for the smooth functioning of such a research organization and for ensuring the sustainability and development of cohort studies. In other words, researchers involved in cohort studies not only conduct statistical analysis using the constructed database and write manuscripts, they also should actively participate in the process of data collection and management and experience the entire process of clinical epidemiology research. Furthermore, they should participate in a series of clinical epidemiology research processes, from conceiving the research questions to specific research planning and protocol development. Through repetition of such practical learning, students will be encouraged to acquire the research execution skills to promote clinical research. Researchers who are skilled in clinical epidemiology research, and who have successfully completed a series of on-the-job training programs, form the most important basis for long-term follow-up of cohorts and addressing new developments.

## Conclusion

In this review, we aimed to elucidate prognostic factors and validate the effects of treatment through the description and analysis of cohort studies conducted among patients with CKD. To extract high-quality evidence from observational databases that can lead to disease prevention and intervention studies, it is necessary to apply appropriate statistical analysis methods. The search for novel disease risk markers is now underway, and risk models incorporating risk markers that stratify specific populations must be created to verify the effectiveness of personalized treatment based on accurate individual risk prediction. For the long-term maintenance and development of cohort studies, in addition to the establishment of research organizations to ensure the quality of research, training of clinical researchers who are familiar with the process of clinical epidemiological research and who have the ability to manage practical affairs is required.
